# Supplementary material for: Sarcopenia Is Associated with Cognitive Impairment Mainly Due to Slow Gait Speed: Results from the Korean Frailty and Aging Cohort Study (KFACS)
Source: Int J Environ Res Public Health. 2019 Apr 27;16(9):1491. doi: 10.3390/ijerph16091491 (PMC6539557; doi:10.3390/ijerph16091491)
Supplement: Supplementary file 1 [file ijerph-16-01491-s001.pdf]

Supplementary data

Table S1. Associations of body composition index, muscle strength, and muscle function with neuropsychological test scores

	Dependent variable	Male			Female		
		Adjusted Model 3 B	95% CI	*p*-value	*Adjusted Model 3 B*	95% CI	*p*-value
Appendicular skeletal muscle mass index, kg/h² *(adjusted for height)*	Mini-Mental State Examination, score	−0.174	−0.394, 0.046	.212	−0.030	−0.296, 0.236	.822
	Word list learning, score	0.036	−0.293, 0.365	.831	0.479	0.094, 0.863	.015
	Word list recall, score	0.030	−0.140, 0.199	.732	0.197	0.000, 0.394	.049
	Word list recognition, score	−0.060	−0.215, 0.094	.446	0.157	−0.023, 0.336	.087
	Trail making test A, s	1.060	−1.803, 3.922	.468	2.337	−2.880, 7.554	.379
	Digit span forward, score	0.023	−0.098, 0.144	.741	0.083	−0.056, 0.222	.242
	Digit span backward, score	−0.076	−0.163, 0.010	.084	−0.026	−0.122, 0.069	.588
	Frontal Assessment Battery, score	−0.205	−0.414, 0.003	.054	−0.215	−0.463, 0.032	.088
Appendicular skeletal muscle mass index, kg/BMI (adjusted for body mass index)	Mini-Mental State Examination, score	−1.246	−2.828, 0.336	.123	−0.016	−2.451, 2.419	.990
	Word list learning, score	−2.211	−4.568, 0.145	.066	0.517	−3.016, 4.050	.774
	Word list recall, score	−0.650	−1.870, 0.570	.296	0.521	−1.285, 2.327	.572
	Word list recognition, score	−0.814	−1.925, 0.298	.151	−1.146	−1.791, 1.499	.862
	Trail making test A, s	−0.687	−21.292, 19.918	.948	15.547	−35.240, 63.334	.523
	Digit span forward, score	0.637	−0.231, 1.504	.150	1.040	−0.233, 2.312	.109
	Digit span backward, score	−0.086	−1.709, 0.537	.788	0.072	−0.802, 0.947	.871
	Frontal Assessment Battery, score	−0.184	−1.688, 1.320	.810	−1.302	−3.571, 0.967	.260
Body fat percentage, %[a]	Mini-Mental State Examination, score	0.039	0.007, 0.071	.018	0.046	0.009, 0.082	.014
	Word list learning, score	0.087	0.039, 0.134	.000	0.036	−0.017, 0.089	.179
	Word list recall, score	0.030	0.005, 0.055	.018	0.020	−0.007, 0.046	.156
	Word list recognition, score	0.024	0.001, 0.047	.037	0.029	0.005, 0.054	.019
	Trail making test A, s	−0.468	−0.886, −0.046	.029	−1.834	−2.538, −1.130	.000
	Digit span forward, score	−0.017	−0.035, 0.001	.060	−0.018	−0.037, 0.001	.062
	Digit span backward, score	0.019	0.006, 0.032	.003	0.011	−0.002, 0.024	.099
	Frontal Assessment Battery, score	0.019	−0.011, 0.050	.218	0.052	0.018, 0.085	.003
Grip strength, kg	Mini-Mental State Examination, score	0.047	0.017, 0.078	.002	0.043	−0.004, 0.090	.076
	Word list learning, score	0.057	0.012, 0.103	.013	0.038	−0.031, 0.106	.760
	Word list recall, score	0.015	−0.009, 0.039	.214	0.026	−0.009, 0.061	.151
	Word list recognition, score	0.016	−0.005, 0.038	.132	0.022	−0.101, 0.054	.181

	Trail making test A, s	−0.831	−0.226, −0.437	.000	−0.592	−1.521, 0.336	.211
	Digit span forward, score	−0.003	−0.020, 0.014	.730	−0.011	−0.036, 0.014	.374
	Digit span backward, score	0.004	−0.008, 0.014	.503	0.018	0.001, 0.035	.041
	Frontal Assessment Battery, score	0.058	0.030, 0.087	.000	0.045	0.001, 0.089	.044
Usual gait speed, m/s	Mini-Mental State Examination, score	0.714	0.061, 1.366	.032	1.010	0.201, 1.819	.014
	Word list learning, score	1.371	0.401, 2.341	.006	2.228	1.059, 3.397	.000
	Word list recall, score	0.573	0.071, 1.076	.025	0.708	0.108, 1.308	.021
	Word list recognition, score	0.599	0.141, 1.056	.010	0.510	−0.037, 1.057	.068
	Trail making test A, s	−12.593	−21.061, −4.125	.004	−26.580	−42.417, −10.744	.001
	Digit span forward, score	0.230	−0.128, 0.588	.207	0.271	−0.154, 0.695	.211
	Digit span backward, score	0.256	−0.001, 0.513	.051	0.100	−0.192, 0.391	.391
	Frontal Assessment Battery, score	1.289	0.673, 1.904	.000	1.101	0.347, 1.854	.004

Notes: Model 3: Adjusted for age and education, smoking status, alcohol intake, Mini Nutritional Assessment Screening score (≤ 11), low physical activity, body mass index, number of comorbidities, depressive symptoms, and self-reported health status (good vs. poor).

B = unstandardized coefficients using multiple linear regression.

[a]Percentage body fat was determined as total fat mass divided by total body mass by dual-energy X-ray absorptiometry.
